# Identification of Candidate B-Lymphoma Genes by Cross-Species Gene Expression Profiling

**DOI:** 10.1371/journal.pone.0076889

**Published:** 2013-10-09

**Authors:** Van S. Tompkins, Seong-Su Han, Alicia Olivier, Sergei Syrbu, Thomas Bair, Anna Button, Laura Jacobus, Zebin Wang, Samuel Lifton, Pradip Raychaudhuri, Herbert C. Morse, George Weiner, Brian Link, Brian J. Smith, Siegfried Janz

**Affiliations:** 1 Department of Pathology, University of Iowa Carver College of Medicine, Iowa City, Iowa, United States of America; 2 Bioinformatics, University of Iowa Carver College of Medicine, Iowa City, Iowa, United States of America; 3 Department of Biostatistics, University of Iowa College of Public Health, Iowa City, Iowa, United States of America; 4 Department of Internal Medicine, University of Iowa Carver College of Medicine, Iowa City, Iowa, United States of America; 5 Holden Comprehensive Cancer Center, University of Iowa Carver College of Medicine, Iowa City, Iowa, United States of America; 6 Department of Biochemistry and Molecular Genetics, University of Illinois at Chicago College of Medicine, Chicago, Illinois, United States of America; 7 Department of Statistics & Actuarial Science, University of Iowa College of Liberal Arts & Sciences, Iowa City, Iowa, United States of America; 8 Laboratory of Immunogenetics, National Institute of Allergy and Infectious Diseases, National Institutes of Health, Rockville, Maryland, United States of America; University of Navarra, Center for Applied Medical Research, Spain

## Abstract

Comparative genome-wide expression profiling of malignant tumor counterparts across the human-mouse species barrier has a successful track record as a gene discovery tool in liver, breast, lung, prostate and other cancers, but has been largely neglected in studies on neoplasms of mature B-lymphocytes such as diffuse large B cell lymphoma (DLBCL) and Burkitt lymphoma (BL). We used global gene expression profiles of DLBCL-like tumors that arose spontaneously in *Myc*-transgenic C57BL/6 mice as a phylogenetically conserved filter for analyzing the human DLBCL transcriptome. The human and mouse lymphomas were found to have 60 concordantly deregulated genes in common, including 8 genes that Cox hazard regression analysis associated with overall survival in a published landmark dataset of DLBCL. Genetic network analysis of the 60 genes followed by biological validation studies indicate *FOXM1* as a candidate DLBCL and BL gene, supporting a number of studies contending that FOXM1 is a therapeutic target in mature B cell tumors. Our findings demonstrate the value of the “mouse filter” for genomic studies of human B-lineage neoplasms for which a vast knowledge base already exists.

## Introduction

Comparative gene expression profiling of malignant tumor counterparts in humans and model organisms such as laboratory rats and mice affords a unique, powerful approach to identify genetic networks that have been conserved in neoplastic cell development over millions of years of evolution. The principal objective of cross-species analyses of human cancer transcriptomes is the discovery of concordantly deregulated genes in corresponding types of cancer in model organisms. Genes of this sort point to common drivers of tumor development or pathways of tumor maintenance, therapy response, acquisition of drug resistance and/or overall outcome. Cross-species comparisons of global gene expression patterns have been successfully employed in the past for gene discovery in liver cancer [1], breast cancer [2], lung cancer [3], prostate cancer [4], intestinal adenoma [5], melanoma [6], rhabdomyosarcoma [7] and, as far as hematopoietic tumors are concerned, T-cell lymphoma [8]. However, this approach has been underutilized in aggressive malignant tumors of B-lymphocytes, such as diffuse large B cell lymphoma (DLBCL), the most prevalent type of non-Hodgkin’s lymphoma in Western countries.

DLBCL has been extensively characterized at the genetic, biochemical and oncogenomic level [9-11], contributing to our current understanding that DLBCL is a heterogeneous disease that (i) is comprised of different histopathologic, genetic and molecular subtypes, (ii) requires different approaches to therapy and patient management and iii) exhibits different outcomes in accordance with BCL2 expression [12], occurrence of *MYC*-activating chromosomal translocations [13], cytogenetic complexity [14] and patient age at diagnosis [15] – to name but a few prognostic parameters. Genetically engineered mouse models in which DLBCL-like neoplasms develop spontaneously in their native immunocompetent microenvironment [16] can make valuable contributions to the rich genetic and biological background on DLBCL, particularly with regard to elucidating mechanisms of tumor development. Mouse models of this sort may also afford a good opportunity to evaluate the hypothesis that comparative gene expression profiling of human-mouse lymphoma counterparts generates new and clinically relevant insight into a type of mature B-lineage tumor for which a vast knowledge base already exists. Here we report the result of a feasibility study to that end.

The study began with a comparative transcriptomic analysis of 9 human DLBCL and 7 B lymphoma tumors that developed in strain C57BL/6 (B6) mice that harbored a targeted insertion of a single copy of a mouse *Myc* cDNA gene into the immunoglobulin heavy-chain (*Igh*) locus. The transgene, dubbed “iMyc” for “inserted” *Myc*, mimics the chromosomal t(8;14)(q24;q32) translocation found in the great majority of human Burkitt lymphoma (BL) and a subset of DLBCL and other B-lineage tumors [17]. This particular study used tumor samples that harbor *Myc* in each of three distinct *Igh* insertion sites [17]. Approximately 70% of B6.iMyc congenic mice develop a type of high-grade, iMyc-driven, B-cell lymphoma (designated iMycBCL) by 12 months of age that recapitulates histopathologic features of aggressive human B lymphoma, such as DLBCL and BL. Cross-species expression profiling of the human-mouse tumor counterparts uncovered 60 concordantly deregulated genes that are referred to below as DMB genes (short for human DLBCL and mouse iMycBCL genes). Eight of these genes were found to be associated with survival of DLBCL patients included in the dataset of Lenz et al. [18], indicating clinical relevance of the DMB 60-gene list. Network analysis of six DMB genes frequently confirmed in studies on the DLBCL transcriptome enabled the discovery of *FOXM1*, the forkhead box M1 transcription factor, as a top candidate lymphoma gene.

Although limited in terms of sample size, the result of this pilot study demonstrated the merit of comparative gene expression profiling of B-lineage tumors across the human-mouse species barrier. Extending this approach to other types of B-cell tumors for which dedicated mouse models have been developed [19] may be warranted.

## Materials and Methods

### Tissue specimens and normal controls

OCT-embedded DLBCL patient samples were from the Biospecimens Core of the University of Iowa/Mayo Clinic Lymphoma SPORE. Healthy volunteer peripheral blood B cells (normal controls) were obtained through the University of Iowa DeGowin Blood Center and isolated using MACS B Cell Isolation Kit II (Miltenyi Biotec). Diffuse, high-grade B220 ^+^ Pax5^+^ iMyc-dependent mouse B-cell lymphoma (iMycBCL) were harvested from enlarged peripheral or abdominal lymph nodes (>75% tumor cells) of strain C57BL/6 mice containing either the iMyc^Eμ^, iMyc^ΔEμ^ or iMyc^Cα^ transgene [17]. Control, splenic B-cells were obtained from inbred C57BL/6 mice and isolated with B220 microbeads (Miltenyi Biotec). The diagnosis of human DLBCL and mouse iMycBCL was independently confirmed by board-certified human (S.S.) and veterinary (A.O.) pathologists. All human samples were de-identified and untraceable to living persons. Mouse studies were performed under protocol 1001004 as approved by the University of Iowa Office of Animal Resources Institutional Animal Care and Use Committee (IACUC).

### RNA processing and microarray analysis

Whole tumor specimens from human or mouse containing >75% neoplastic cells were ground and processed for microarray hybridization. Frozen human and mouse tissue (1–3 mm × 1–3 mm) was submerged in liquid nitrogen in a ceramic mortar, ground to powder, and immediately processed for mRNA isolation using the PerfectPure RNA Tissue Kit (5 PRIME, Hannover, Germany) for total human RNA or the Trizol reagent (Invitrogen, Carlsbad, CA) for total mouse RNA. Gene expression profiling (GEP) was performed on Affymetrix GeneChip Human Genome U133 Plus 2.0 Array (HG U133) and Mouse Genome 430 2.0 arrays (MG 430), respectively.

For human samples, RNA quality was assessed using the Agilent Model 2100 Bioanalyzer (Agilent Technologies, Palo Alto, CA). Total RNA (5 µg) was processed for use on the microarray using the Affymetrix GeneChip one-cycle target labeling kit (Affymetrix, Inc., Santa Clara, CA). The resultant biotinylated cRNA was fragmented and hybridized to the GeneChip Human Genome U133 Plus 2.0 Array (HG U133; Affymetrix, Inc.) to analyze over 47,000 human transcripts. Arrays were washed, stained, and scanned using the Affymetrix Model 450 Fluidics Station and Affymetrix Model 3000 scanner (7G upgrade) at the University of Iowa DNA Core Facility (Iowa City, IA). Expression values were generated by the Micro Array Suite (MAS) v. 5.0 software within the GeneChip operating software (GCOS) v 1.4.

For mouse samples, 50 ng total RNA was converted to cDNA, amplified by SPIA using the Ovation RNA Amplification System v2, and purified using a QIAGEN MinElute Reaction Cleanup column according to modifications from NuGEN. SPIA-amplified cDNA (3.75 µg) was fragmented (average fragment size = 85 bases) and biotin labeled using the NuGEN FL-Ovation cDNA Biotin Module v2. The resulting biotin-labeled cDNA was mixed with Affymetrix hybridization buffer, placed onto Mouse Genome 430 2.0 arrays (MG 430), and incubated at 45°C for 18 hrs (60 rpm rotation) in an Affymetrix Model 640 Genechip Hybridization Oven. Arrays were then washed, stained with streptavidin-phycoerythrin (Molecular Probes, Inc., Eugene, OR) and signal-amplified with anti-streptavidin antibody (Vector Laboratories, Inc., Burlingame, CA) using the Affymetrix Model 450Fluidics Station. Arrays were scanned and expression values generated as described for human samples.

HG U133 and MG 430 array data were imported into Partek GS (v6.3) and normalized as separate batches using default settings for robust multi-averaging (RMA). Variability of the data was assessed by principal component analysis (PCA) per respective species (Figure **S1**). Normal and tumor samples were compared using ANOVA. False discovery rate (FDR) correction relied on default settings for the step-up method in Partek. For both species, FDR of 0.01 was used and two separate lists containing genes highly likely to be differentially expressed were generated. Using homology associations from Affymetrix annotations, the mouse probe sets that were significantly changed were associated with their human counterparts. Genes exhibiting significant concordant change in mouse and human tumors were identified. Array data are available under the following gene expression omnibus (GEO) accession number: GSE44337.

### Quantitative RT-PCR

Total RNA was reverse transcribed to generate cDNA using either a First Strand cDNA synthesis kit (Roche) kit or Superscript III First Strand Synthesis SuperMix (Invitrogen). Primers and probes were purchased from Integrated DNA Technologies (IDT, Coralville, IA). Sequences provided in Table **S1**. Quantitative RT-PCR (qPCR) was performed using Taqman® Gene Expression Master Mix (Applied Biosystems, Foster City, CA) on an ABI 7000 or 7900HT at the University of Iowa DNA Core Facility. Data were analyzed using ABI Sequence Detection Software v1.2 or v2.3, Microsoft Excel and GraphPad Prism (v5). Statistics tested the null hypothesis that the expression level did not differ between normal and tumor tissue using a two-sided, non-parametric Mann-Whitney with a 95% confidence interval.

### Cell lines and treatments

Burkitt lymphoma cell lines (Daudi, DG75, Raji, Ramos) were obtained from ATCC (Manassas, VA). Dawo was derived in our laboratory from the pleural effusion of a patient with an aggressive B lymphoma. All human DLBCL cell lines (BJAB, OCI-Ly7, SU-DHL-4, SU-DHL-6, HBL1, TMD8, OCI-LY3, OCI-Ly10) were kindly provided by Dr. R. Eric Davis (University of Texas, Houston, TX) [20]. *O*CI-Ly3 and OCI-Ly10 were cultured in IMDM supplemented with 20% human plasma, HEPES, L-glutamine, penicillin/streptomycin, and 2-mercaptoethanol. Daudi, DG75, Raji, Ramos, BJAB, OCI-Ly7, SU-DHL-4, SU-DHL-6, HBL1, and TMD8 were cultured in RPMI supplemented with 10% FBS, L-glutamine, penicillin/streptomycin, HEPES and sodium pyruvate.

Cells were treated with thiostrepton (Sigma-Aldrich, St. Louis, MO) and ARF-peptide or a mutated peptide [21] (Genemed Synthesis, Inc., San Antonio, TX) as detailed in the Results.

### Cell metabolic activity, viability, apoptosis, DNA Content

Cell metabolic activity and viability were examined using CellTiter 96® Aqueous One Solution Cell Proliferation Assay (MTS-based) (Promega), CellTiter-Glo® Luminescent Cell Viability Assay (Promega), and/or Guava ViaCount® (EMD Millepore) 24 hours after seeding at 4 x 10^5^ c/ml. A BD FACSCalibur flow cytometer was used to determine active apoptosis by AnnexinV and propidium iodide (PI) cell staining (BD Phamingen), and DNA content by PI in a hypotonic lysis buffer as before [22].

### Online databases

Oncomine 4.4 (https://www.oncomine.org/resource/login.html) was used for comparisons to other lymphoma GEPs using parameters discussed in Results [23]. STRING 9.0 (http://string-db.org/) was employed for network analysis as described in the text [24]. These data were imported into Cytoscape [25] to generate the pathway diagram. The National Cancer Institute Pathway Interaction Database (NCI-PID; http://pid.nci.nih.gov/) “batch query” feature was used for pathway analysis of the NCI-Nature curated data sources [26]. DMB gene ontology was determined using DAVID (http://david.abcc.ncifcrf.gov/home.jsp).

### Survival statistics

Associations between gene expression and overall survival were individually estimated and assessed for statistical significance using Cox regression models. Overall survival was defined as time survived from treatment to death. Subjects alive at the end of the study follow-up were treated as censored observations in the survival analysis. Results are reported as the relative rates of death associated with one-unit increases in expression level (hazard ratios), along with 95% confidence intervals. Statistical tests were performed to assess the significance of treatment-specific hazard ratios as well as between-treatment differences in hazard ratios. All tests were two-sided and assessed for significance at the 5% level.

## Results

### Human DLBCL and mouse iMycBCL contain concordantly deregulated genes

**Figure 1:**
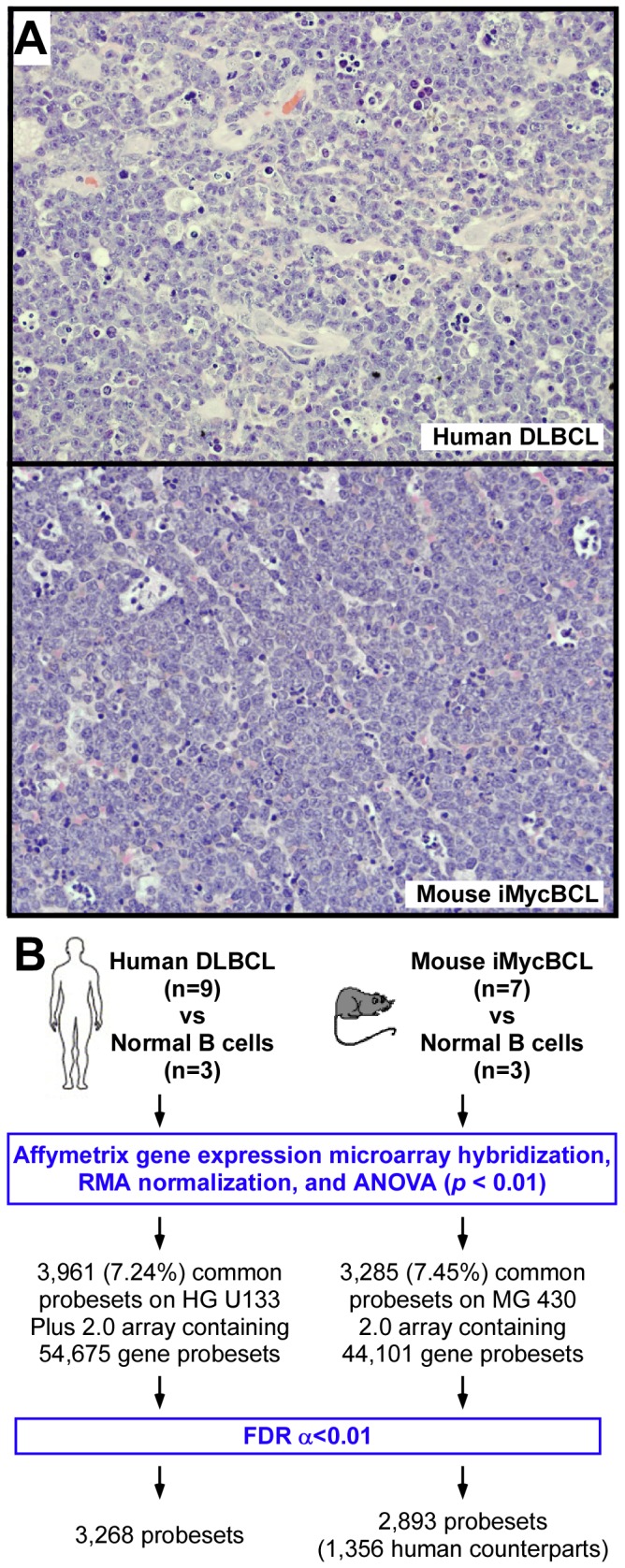
Global gene expression profiles of human and mouse B lymphoma contain an abundance of deregulated genes. (A) Representative tissue sections of human DLBCL (top) and mouse iMycBCL (bottom) stained with hematoxylin and eosin. The normal lymphoid tissue structure is effaced in both species by sheets of medium to large tumor cells that contain scant cytoplasm and round to polygonal nuclei with one to two nucleoli. Tingible body macrophages that harbor apoptotic bodies are abundant. Microscopic slides were read using an Olympus BX-51 light microscope equipped with UPLSAPO objectives (Olympus). The light temperature of the microscope bulb varied between 3000 and 3400 K. Imaging medium was air. Images were acquired with the help of a 40x objective (0.95 numerical aperture), DP2 digital camera (Olympus), and DP2-BSW imaging software (Olympus). Images were saved as TIF (tagged image file) data files and enhanced with respect to brightness, contrast and color balance using the Adobe Photoshop CS2 Version 9.0.2 software (Adobe Systems Inc). (B) Flow chart of global gene expression analysis of human and mouse lymphoma counterparts carried out in parallel and using the same statistical parameters (RMA, robust multi-averaging; ANOVA, analysis of variance; FDR, false discovery rate). Gene expression profiles of human DLBCL and normal B cells were compared on HG U133 microarrays. Gene expression profiles of mouse iMycBCL and normal B cells were compared on MG 430 microarrays.

To evaluate gene expression changes in malignant B-cell lymphoma across human and mouse, 9 human DLBCL and 7 mouse iMycBCL were selected for genome-wide expression profiling on Affymetrix microarrays. Figure **1A** depicts one representative hematoxylin and eosin-stained tissue section each of human and mouse lymphomas, showing their histological similarity. Peripheral blood B cells from 3 healthy donors and B220^+^ splenocytes from 3 inbred C57BL/6 mice served as controls for the human and mouse tumors, respectively. Comparison of RMA-normalized global gene expression profiles (GEP) of DLBCL and normal human B cells using ANOVA (*p* < 0.01) demonstrated significant differences in 3,961 of 54,675 (7.24%) probesets, with 3,268 (82.5%) remaining at a false discovery rate (FDR) of 1% ( < 0.01; Figure **1B** **left**). ANOVA of iMycBCL and normal mouse B cells revealed significant differences in 3,285 of 44,101 (7.45%) probesets, with 2,893 (88.1%) passing the FDR 0.01 filter (Figure **1B** **right**). Less than half (1,356; 46.9%) of the mouse genes represented by the 2,893 differential probesets had an identically named, annotated human counterpart, a restrictive comparison. The results showed that the fraction of differentially regulated genes in malignant versus normal B-lymphocytes is similar in both species: 5.98% (3,268/54,675) in human and 6.56% (2,893/44,101) in mouse.

**Figure 2:**
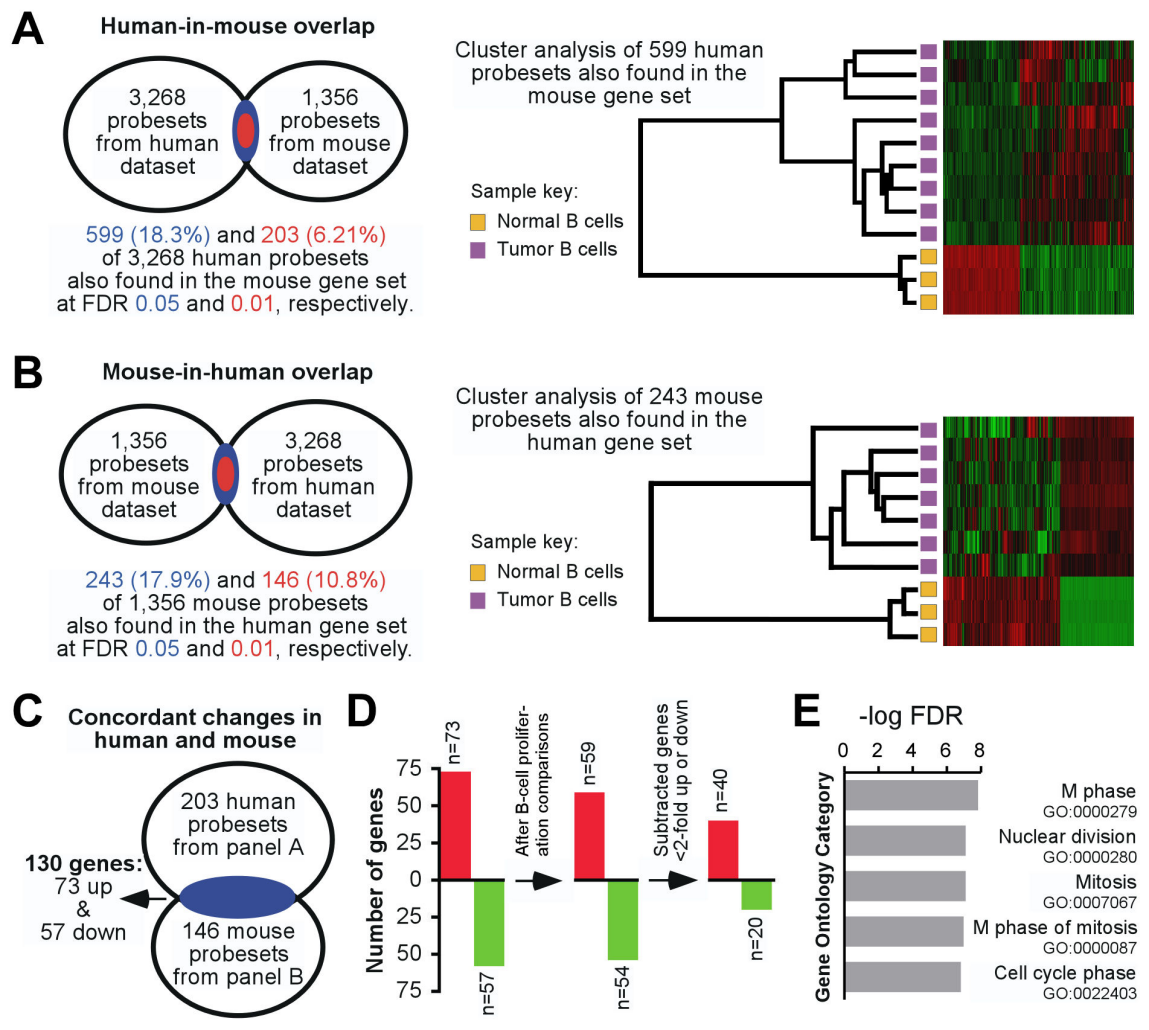
Stringent cross-species comparison of gene expression changes in B-cell lymphoma discovers 60 concordantly deregulated genes, designated DMB (DLBCL/iMycBCL). (A) The Venn diagram on the left shows the degree of human-in-mouse overlap of gene probesets found to be significantly variable in both species. Results at FDR threshold values of 5% and 1% are indicated in blue and red, respectively. A heat map of unsupervised cluster analysis of matching human-mouse gene sets at FDR 0.05 is depicted on the right. (B) Degree of mouse-in-human overlap of significantly variable gene probesets in both species, using the same approach as in panel A. Panels A and B depict reciprocal results. (C) Venn diagram indicating that overlapping gene sets from panels A and B (FDR 0.01 in both cases) represent 130 concordantly deregulated genes in human DLBCL and mouse iMycBCL. (D) Diagrammatic representation of two filtering steps that narrowed the gene list from 130 genes to 60 genes. The genes eliminated in this process are indicated in Table S1. (E) Column diagram indicating the top five gene ontology (GO) categories for the 130 concordantly deregulated genes from panels C and D left. GO categories were determined using DAVID. Pathway names and numbers are shown to the right.

Differentially expressed genes in human and mouse tumors exhibited partial overlap. The degree of human-in-mouse overlap is indicated schematically in Figure **2A** **left**, showing that 599 (18.3%) and 203 (6.21%) of the 3,268 human probesets were also different in iMycBCL at FDR 0.05 and 0.01, respectively. Unsupervised cluster analysis of these 599 probesets resulted in the dendrogram and heat map presented in Figure **2A** **right**. The level of the reciprocal mouse-in-human overlap, shown in Figure **2B**, was similar: 17.9% (243 of 1,356; FDR<0.05) and 10.8% (146 of 1,356; FDR<0.01) of iMycBCL probesets were significantly different in human DLBCL. Further analysis of the species-overlapping gene pool at FDR 0.01 revealed 130 annotated genes (Figure **2C**), 73 genes up regulated (98 human probesets) and 57 genes down regulated (85 human probesets), in lymphoma compared to normal B cells (Figure **2D** **left**; Table **S2** lists all 183 human and mouse probesets). A gene ontology search using DAVID [27], showed that a significant number of the 130 genes participate in the cell cycle, particularly mitosis (Figure **2E**). To examine whether a majority of these differentially expressed genes were also differentially expressed between resting and activated, proliferating states in normal B cells, the 130-gene list was compared to two other GEP studies that contrasted resting to highly proliferative, activated B cells. Differentially expressed genes from resting to *in vitro* activated B cells (GSE6136) [28] or quiescent, naïve to germinal center B cells (GSE4142) [29] (Table **S3**) were identified using the same statistical criteria applied to our datasets. Only 17 overlapping genes were identified (13%), not the majority (4 in GSE6136 and 13 in GSE4142; Figure **2D** **center**; see Table **S2** for details). The genes associated with activated, proliferating normal B cells were eliminated from further consideration to focus on genes identified in mouse iMyc and human DLBCL tumors. Remaining genes were still significantly enriched for those associated with proliferation (by DAVID, not shown), but our analysis suggests that the majority of genes we identified by comparing tumor to normal tissue are different from genes identified by comparing quiescent to highly proliferative normal B cells. The last layer of stringency eliminated 52 genes that were less than two-fold up (n = 20) or down (n = 32) in human-mouse lymphoma counterparts (Figure **2D** **right**), resulting in 60 genes (40 up and 20 down), referred to as DMB genes (human DLBCL & mouse iMyc B-cell lymphoma genes) henceforth, listed in Table **1**.

**Table 1:** Differentially expressed genes in human DLBCL and mouse iMycBCL, designated DMB genes.

	**Human**			**Mouse**		
**Gene Symbol**	**Probeset ID**	***p* value**	**Fold Change**	**Probeset ID**	***p* value**	**Fold Change**
**AURKB**	209464_at	1.00E-03	**8.94**	1451246_s_at	2.80E-03	**4.54**
**BCAT1**	226517_at	9.30E-04	**33.8**	1450871_a_at	2.40E-03	**10.24**
	225285_at	1.70E-03	**22.7**			
**BIRC5**	202095_s_at	1.30E-04	**12.63**	1424278_a_at	1.60E-03	**3.69**
	202094_at	6.00E-03	**3.34**			
**BOLA3**	227291_s_at	9.40E-04	**3.17**	1433970_at	5.30E-03	**2.13**
**BUB1B**	203755_at	1.30E-04	**20.2**	1447363_s_at	1.10E-03	**7.06**
**CCDC99**	221685_s_at	1.40E-03	**2.9**	1424971_at	6.90E-03	**2.44**
**CCNA2**	203418_at	1.10E-05	**12.82**	1417911_at	1.30E-03	**3.62**
	213226_at	2.10E-05	**6.71**			
**CCNB2**	202705_at	1.70E-05	**22.67**	1450920_at	7.40E-03	**2.98**
**CCT3**	200910_at	1.30E-03	**2.36**	1459987_s_at	5.70E-03	**2.38**
**CDK1**	203213_at	1.00E-05	**42.72**	1448314_at	3.00E-04	**5.39**
	210559_s_at	7.50E-06	**25.98**			
	203214_x_at	1.40E-05	**19.09**			
**CENPA**	204962_s_at	5.40E-04	**10.2**	1450842_a_at	2.00E-03	**2.9**
**CKS1B**	201897_s_at	6.20E-03	**2.96**	1448441_at	1.00E-03	**5.14**
**CKS2**	204170_s_at	1.10E-04	**15.51**	1417458_s_at	2.30E-03	**3.22**
**CLCF1**	219500_at	2.90E-04	**-2.14**	1450262_at	4.50E-04	**-2.08**
**COBLL1**	203641_s_at	8.70E-03	**-8.74**	1458097_at	1.80E-04	**-3.1**
	229598_at	1.70E-03	**-2.17**			
**CTPS**	202613_at	9.30E-04	**5.71**	1416563_at	1.10E-04	**3.98**
**DOCK11**	238356_at	1.80E-04	**-2.39**	1443467_at	6.50E-04	**2.34**
**ESPL1**	38158_at	1.00E-03	**4.42**	1433862_at	6.00E-04	**4.79**
	204817_at	4.60E-03	**3.23**			
**FABP5**	202345_s_at	3.50E-04	**12.47**	1416022_at	5.60E-03	**20.82**
**FAM65B**	206707_x_at	2.20E-04	**-7.34**	1460555_at	1.30E-03	**-8.93**
**FKBP1A**	200709_at	4.70E-04	**2.61**	1456196_x_at	1.70E-03	**2.56**
	214119_s_at	7.90E-04	**2.27**			
	210186_s_at	8.20E-03	**2.03**			
**FOSB**	202768_at	3.40E-05	**-11.83**	1422134_at	5.70E-04	**-5.19**
**GMNN**	218350_s_at	6.30E-04	**10.78**	1417506_at	3.00E-04	**2.89**
**HECA**	230529_at	8.70E-05	**-13.57**	1434478_at	3.20E-03	**-2.61**
**HMGA1**	206074_s_at	6.70E-03	**3.87**	1416184_s_at	3.20E-03	**3.75**
**HMGB3**	203744_at	2.40E-03	**5.25**	1416155_at	6.90E-03	**3.52**
**HSPD1**	200807_s_at	3.50E-04	**2.69**	1426351_at	2.20E-03	**2.58**
	200806_s_at	5.10E-03	**2.41**			
**JMJD1C**	228793_at	1.70E-03	**-2.88**	1448049_at	3.80E-04	**-3.14**
	221763_at	2.80E-03	**-2.3**			
**KIF18B**	222039_at	1.30E-04	**12.7**	1453226_at	1.10E-03	**3.13**
**KIF20A**	218755_at	3.80E-04	**6.51**	1449207_a_at	4.70E-03	**4.68**
**LDHA**	200650_s_at	5.10E-04	**3.65**	1419737_a_at	1.90E-03	**2.74**
**LGALS3**	208949_s_at	2.00E-03	**-29.33**	1426808_at	8.60E-03	**-3.48**
**MAP3K1**	214786_at	1.00E-04	**-11.89**	1443540_at	2.90E-03	**-4.19**
	225927_at	4.80E-03	**-5.03**			
**MARCH1**	1562338_at	1.30E-05	**-2.33**	1440209_at	4.40E-04	**-11.5**
**MRPS17**	218982_s_at	1.70E-03	**3.42**	1453728_a_at	4.10E-03	**2.16**
**NDC80**	204162_at	5.10E-05	**10.97**	1417445_at	8.60E-03	**2.5**
**NDUFB6**	203613_s_at	9.90E-03	**2.09**	1434057_at	2.10E-03	**2.36**
**NEK2**	204641_at	1.40E-04	**11.97**	1437580_s_at	4.40E-03	**3.01**
**ORC6L**	219105_x_at	4.50E-03	**3.5**	1417037_at	1.40E-03	**2.49**
**PBK**	219148_at	1.40E-03	**19.23**	1448627_s_at	1.30E-03	**6.99**
**PBXIP1**	207838_x_at	6.80E-03	**-2.02**	1451132_at	1.70E-03	**-3.15**
**PDE7A**	224046_s_at	8.20E-03	**-2.54**	1458218_s_at	6.40E-03	**-3.91**
**PFDN6**	222029_x_at	1.70E-03	**2.7**	1415744_at	5.00E-03	**2.16**
	233588_x_at	9.30E-03	**2.28**			
**PHC3**	215521_at	6.60E-03	**-2.56**	1455312_at	6.50E-03	**-3.08**
	226508_at	2.30E-03	**-2.23**			
**RABEP2**	74694_s_at	5.70E-03	**-2.89**	1440795_x_at	4.10E-03	**-4.74**
	219057_at	1.70E-03	**-2.09**			
**RACGAP1**	222077_s_at	1.50E-05	**12.79**	1451358_a_at	2.10E-03	**5.11**
**RIN3**	60471_at	1.00E-03	**-4.6**	1434684_at	7.60E-03	**-4.58**
	219456_s_at	8.30E-04	**-2.06**			
	219457_s_at	1.30E-03	**-3.46**			
**SAP30**	204900_x_at	7.90E-03	**4.65**	1417719_at	4.10E-03	**3.92**
**SFXN1**	230069_at	7.90E-04	**3.75**	1417560_at	4.50E-03	**2.04**
	218392_x_at	1.40E-03	**2.11**			
**SPAG5**	203145_at	2.30E-04	**6.81**	1433893_s_at	6.00E-03	**4.73**
**TMEM55B**	225287_s_at	8.30E-03	**-2.03**	1454797_at	9.10E-03	**-2.94**
**TOP2A**	201292_at	1.70E-06	**29.72**	1454694_a_at	2.40E-03	**2.33**
	201291_s_at	1.10E-04	**11.93**			
**TTK**	204822_at	8.80E-04	**8.18**	1449171_at	1.60E-05	**6.61**
**VAMP2**	201557_at	4.80E-05	**-2.99**	1420834_at	4.30E-03	**-2.01**
	201556_s_at	1.40E-03	**-2.14**			
**VDAC1**	212038_s_at	5.10E-04	**3.12**	1437947_x_at	4.90E-05	**2.61**
	217140_s_at	6.50E-03	**2.58**			
**WDFY2**	1560112_at	1.70E-05	**-5.49**	1434517_at	1.30E-04	**-3.8**
	227490_at	2.90E-03	**-2.09**			
**YPEL3**	232077_s_at	1.70E-03	**-4.15**	1426624_a_at	4.90E-04	**-4.13**
	223179_at	3.70E-03	**-2.98**			
**ZMYM5**	215948_x_at	2.70E-04	**-2.75**	1445543_at	6.80E-05	**-4.04**
	206652_at	7.30E-04	**-2.29**			
**ZMYM6**	227594_at	2.30E-03	**-2.74**	1438685_at	7.00E-05	**-3.11**
	219925_at	1.10E-03	**-2.24**			
**ZWILCH**	218349_s_at	5.40E-03	**3.79**	1416757_at	9.00E-04	**3.34**
	222606_at	7.60E-04	3.51			

**Figure 3:**
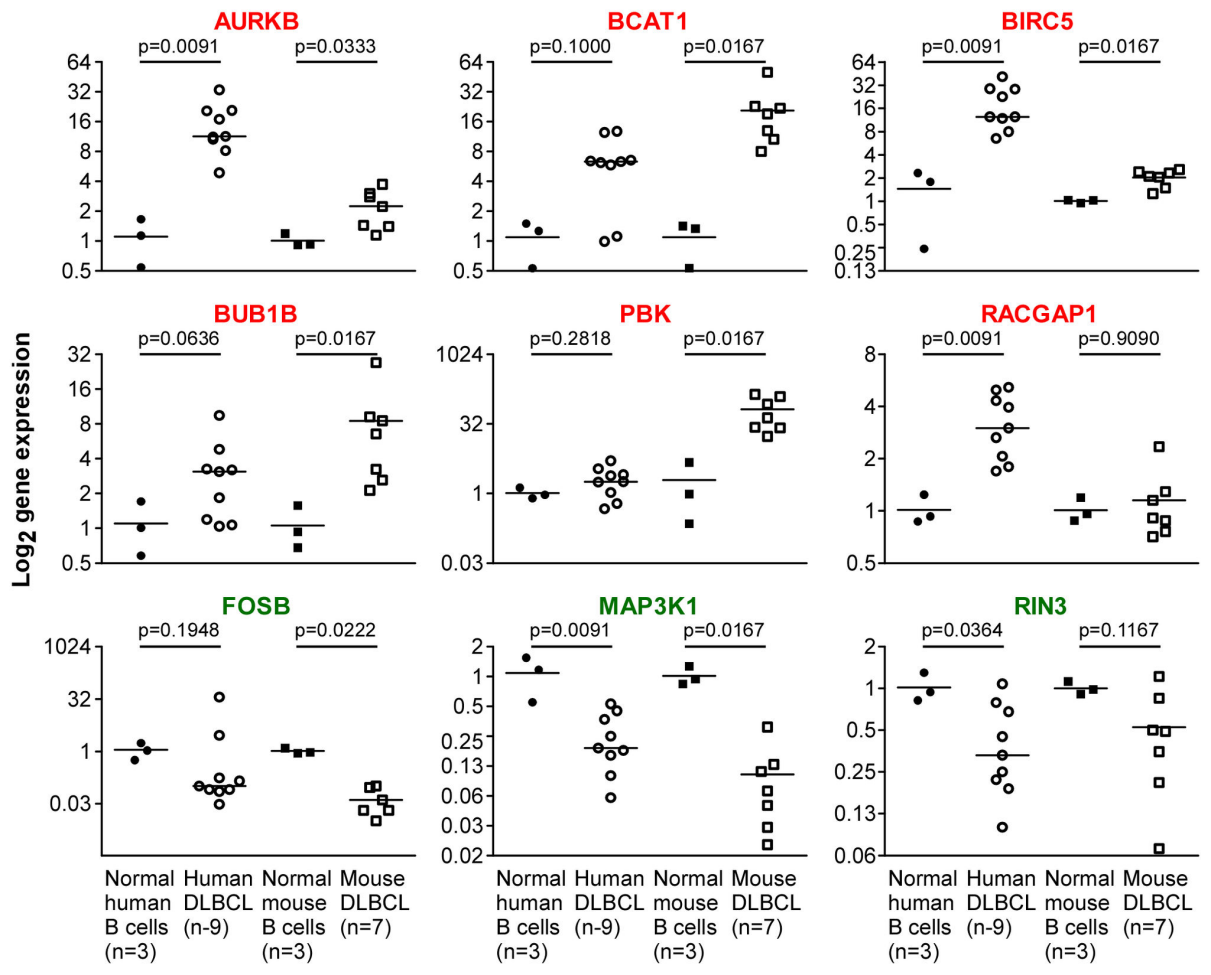
Validation of microarray data using quantitative PCR. Expression levels of DMB genes found to be up regulated (red; top and center rows) or down regulated (green; bottom row) on microarrays were determined with the assistance of qPCR (ΔΔCT method) in human (circle) and mouse (square) lymphoma (open) counterparts. Normal B cells (closed) were included for comparison. *HPRT1* and *Hprt* were used as internal reference genes for human and mouse samples, respectively. Median gene expression levels are indicated by horizontal lines. Statistical analysis relied on the Mann-Whitney test.

To validate the microarray-based expression results, 9 human-mouse gene pairs from the 60-gene list were non-randomly chosen for analysis by quantitative RT-PCR (qPCR). These included six upregulated (Figure **3** **top and center rows**) and 3 downregulated genes (Figure **3** **bottom row**) found in the upper 50% by fold-change in both species. *AURKB*, *BIRC5*, and *MAP3K1* reached statistical significance (*p* < 0.05) in both species. The results were significant in either human but not mouse for *RACGAP1* and *RIN3*, or mouse but not human for *BCAT1, BUB1B, PBK*, and *FOSB*. Although this outcome was not fully consistent with gene chip results (likely due to small sample numbers, uneven representation of tumors and controls and/or outlier values), the qPCR dataset clearly trended with the microarray data. Median expression levels were invariably higher for *AURKB, BCAT1, BIRC5, BUB1B, PBK* and *RACGAP1* and lower for *FOSB, MAP3K1* and RIN3 by qPCR in lymphoma versus normal B cell samples in both species. These findings supported the contention that the DMB 60-gene list shown in Table 1 represents a set of stringently filtered candidate genes that have been phylogenetically conserved, over millions of years of mammalian evolution, in genetic pathways governing neoplastic B-lymphocyte maintenance.

### DMB genes predict overall survival of patients with DLBCL

**Figure 4:**
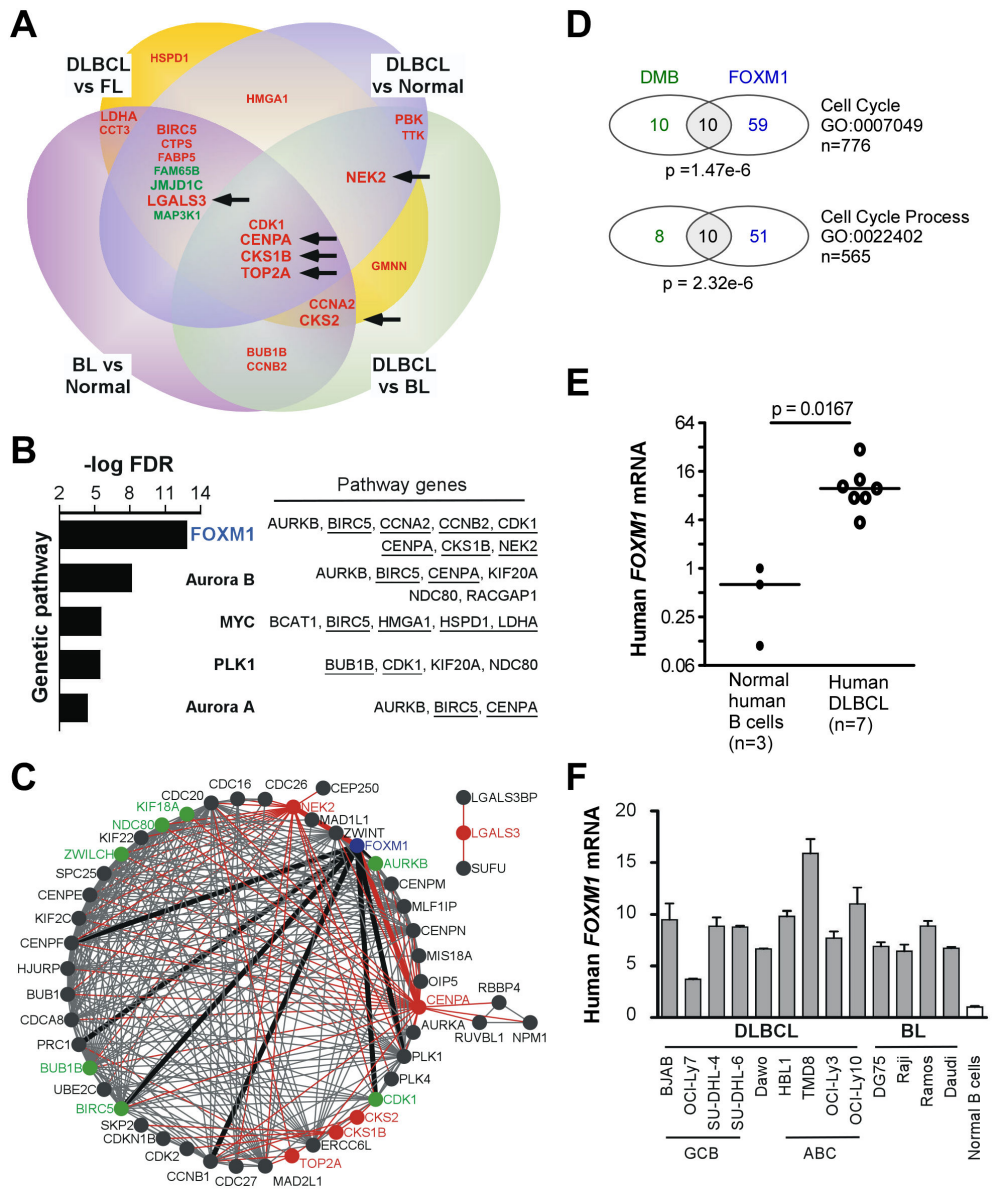
DMB genes are part of the FOXM1 genetic network. (A) Venn diagram displaying symbols of 23 of 60 DMB genes found to be more than 2-fold up (red) or down (green) regulated (*p* < 0.01) in at least 2 of 7 independent GEP studies on human lymphoma within the 4 comparison categories listed (see Table S3 for more details). Increasing font size of the gene symbol relates to an increase in the number of independent studies (2, 3, or 4) in which the gene was found to be differentially expressed across all categories. Black arrows denote the 6 genes found in 4 independent studies.(B) Column diagram indicating the result of Pathway Interaction Database (PID) analysis of the 60DMB genes. The 5 most significant pathways are shown. Genes contained in these pathways are shown to the right. Those included in panel A are underlined. (C) Network diagram representing the result of STRING analysis after expansion to a total of 50 nodes, using the 6 top genes from panel A (nodes and connecting edges indicated in red) as the only input genes. Five of 6 genes are part of a major genetic network that includes FOXM1 (node indicated in blue; edges darkened and thickened for emphasis) and 7 additional DMB genes (indicated in green). One of 6 genes, *LGALS3*, interacts with two other genes outside the FOXM1-associated network. (D) Venn diagrams showing the number of genes (by gene symbol) that overlap between DMB and FOXM1 target genes within the gene ontology (GO) category listed to the right. FOXM1 target genes were taken as defined by Chen et al. [37]. The given p-value indicates that FOXM1 genes are overrepresented in the DMB set and is the result of a Fisher’s Exact test to compare the proportions with a significance level set at p<0.0001. (E) qPCR result unequivocally demonstrating elevated *FOXM1* mRNA in DLBCL cells (11.6 ± 3.22; open) relative to normal B cells (0.580 ± 0.258; closed). Median gene expression level is indicated by horizontal lines. Statistical analysis relied on the Mann-Whitney test. (F) Elevation of *FOXM1* mRNA in DLBCL and BL cells compared to normal B cells, using qPCR as measurement tool. The classification of DLBCL lines as germinal center B cell (GCB) or activated B cell (ABC) type is indicated at the bottom. The assignment of Dawo to either of these categories is unclear.

The web-based cancer database ONCOMINE [23] was employed to evaluate whether DMB genes had been previously identified in GEP studies of aggressive human B lymphomas. Six published datasets on gain of aggressive disease features in DLBCL and gene expression changes in DLBCL compared to follicular lymphoma (FL), Burkitt lymphoma (BL) and normal B cells were found in ONCOMINE [30-35]. Only genes with at least two-fold differential expression and one percent or less probability that the up or down regulation occurred by chance (p ≤ 0.01) were considered. One additional dataset not found in ONCOMINE, Andreasson et al. [36] used the same array platform as our studies and examined gene expression changes occurring when low-grade FL undergoes transformation to high-grade DLBCL, and was thus included using the authors’ own statistical criteria (FDR 0.05). Table **S4** shows that 23 of 60 (38.3%) DMB genes were identified in at least two of the seven independent studies described above. The Venn diagram presented in Figure **4A** indicates the frequency of detection of these genes within 4 different categorical comparisons of GEPs. Six of 23 genes (*CENPA, CKS1B, CKS2, LGALS3, NEK2*, *TOP2A*) were found in at least 4 independent datasets (indicated by large font size and black arrows), and 6 other genes were identified in 3 datasets (*BIRC5, CCNA2, CDK1, JMJD1C, LDHA* and *PBK*). The repeated discovery of these genes in studies on human DLBCL and related B-cell neoplasms supported the contention that DMB genes may be clinically relevant.

To begin to examine this question, the landmark DLBCL dataset provided by Lenz et al. [18] was used to assess whether there was a possible association between DMB gene expression levels and overall survival of lymphoma patients. Because of inherent variability between the two treatment groups, CHOP (cyclophosphamide, doxorubicin, vincristine, prednisone) and R-CHOP (CHOP plus rituximab), they were examined separately (Table **S5**). A significance level of 5% (p < 0.05) was used for this analysis. The expression level of 8 of 60 genes (13%) was significantly associated with survival within at least one of the treatment groups: CCT3, CLCF1, COBLL1, CTPS, FABP5, HSPD1, MRPS17 and NDC80 (Table **2**). Each of these genes exhibited consistency between increased probability of death with increasing expression of an up regulated gene or, conversely, decreased probability of death with increasing expression of a down regulated gene, as well as a similar trend in both treatment groups. *CCT3, CTPS, FABP5*, and HSPD1 are also included in the 23-gene set shown in Figure 4A, whereas *CLCF1, COBLL1, MRPS17*, and *NDC80* are not. Based on the observed 8 out of 60 significant genes, a 95% confidence interval for the proportion of significant genes is (0.06, 0.25), which excludes the 5% percent that would be expected by chance and indicates that the observed proportion is more than just chance. This result supported the idea that comparative gene expression profiling of human-mouse lymphoma counterparts may uncover candidate genes that are clinically relevant (e.g., as outcome prognosticators or therapeutic targets) yet difficult to identify when the research effort is confined to human cancer.

**Table 2:** DMB genes individually associated with survival of DLBCL patients.

		**CHOP**			**R-CHOP**			
**Gene Symbol ^1^**	**Affymetrix ID**	**HR ^2^**	**95% CI^3^**		**HR ^2^**	**95% CI^3^**		***p* value** ^4^
**CCT3**	200910_at	1.686*	1.057	2.689	1.682	0.999	2.832	0.9944
CLCF1	219500_at	0.846	0.685	1.045	0.736*	0.61	0.889	0.3366
COBLL1	203641_s_at	0.86*	0.767	0.963	0.976	0.872	1.092	0.1204
COBLL1	229598_at	0.855*	0.76	0.961	0.849*	0.738	0.976	0.9388
**CTPS**	202613_at	1.451*	1.007	2.091	1.476*	1.034	2.106	0.9475
**FABP5**	202345_s_at	1.144	0.836	1.566	1.468*	1.081	1.995	0.2645
**HSPD1**	200806_s_at	1.267	0.739	2.174	1.886*	1.115	3.19	0.3012
**HSPD1**	200807_s_at	1.12	0.88	1.425	1.614*	1.059	2.459	0.1401
**MRPS17**	218982_s_at	1.264	0.879	1.818	1.787*	1.18	2.707	0.2186
**NDC80**	204162_at	1.153	0.848	1.568	1.54*	1.052	2.255	0.2464

^1^ Bold and normal indicates upregulated and downregulated by gene expression profiling, respectively.

^2^ Hazard ratio. Values indicated by asterisk are statistically significant.

^3^ Confidence interval.

^4^ Comparison of CHOP to R-CHOP.

### Genetic interaction maps of DMB genes point to FOXM1 as a candidate lymphoma gene

To interpret the DMB gene list at the systems biology level of genetic network analysis, both DMB genes and the 23 genes included in Figure 4A/Supplemental Table S3 were interrogated with the assistance of the NCI Pathway Interaction Database (NCI-PID). This led, unexpectedly, to the discovery of FOXM1 as the most highly enriched pathway (Figure **4B**). Indeed, 7 of the 23 (30.4%) genes included in Figure 4A are part of the FOXM1 transcription factor network: *BIRC5, CCNA2, CCNB2, CDK1, CENPA, CKS1B* and *NEK2*. NCI-PID analysis also implicated the Aurora kinase A and B pathways and, unsurprisingly, the MYC pathway. Next, the STRING bioinformatics tool [24], which permits the generation of association maps from a small number of input genes, was used to consider the genetic context of the 6 genes identified in at least four independent GEP studies (see Figure 4A black arrows). We hypothesized that expanding these genes to a wider network could enable the recognition of additional DLBCL candidate genes that may not be part of the DMB 60 but on which the 6 genes might depend. Figure **4C** presents the result of network expansion to 50 nodes, relying on network associations *with the highest confidence* based on co-expression, curated pathway databases and/or experimental evidence. Raw data for the network are in Table **S6**. *CENPA, CKS1B, CKS2, NEK2* and *TOP2A* but not *LGALS3* formed an interaction network including *FOXM1*. These results implicated *FOXM1* in the lymphomas included in this study.

Because 20 of the 60 DMB genes (one-third) are associated with the cell cycle by gene ontology (GO:0007049), we wished to determine whether FOXM1 was simply identified because there is an overrepresentation of cell cycle genes in the DMB list, or whether there was an enrichment of cell cycle-related FOXM1 target genes among the DMB genes. To do so, the proportion of FOXM1 target genes, defined in ChIP-seq studies by Chen et al. [37], associated with the cell cycle was compared to the proportion of cell cycle-associated FOXM1 target genes in the DMB 60-gene list. This was done using two related but independent GO terms, GO:0007049-cell cycle and GO:0022402-cell cycle process, by Fisher’s Exact test with a significance level set at p< 0.0001. In both cases there was a significant enrichment of FOXM1 target genes in the cell cycle-related DMB gene list (Figure **4D**), indicating that FOXM1 was not simply identified because a significant proportion of DMB genes are cell cycle genes.

Sufficient amounts of RNA were left over after microarray and validation analyses from 7 human DLBCL samples to employ qPCR for determination of *FOXM1* levels. Figure **4E** demonstrates that the median *FOXM1* expression in lymphoma was about 20-fold elevated compared to normal B cells (*p* = 0.0167). Similar results were obtained with commonly used DLBCL and BL cell lines (Figure **4F**). All 9 DLBCL and all 4 BL lines harbored *FOXM1* levels that were, respectively, 9.1 ± 3.3 fold and 7.4 ± 1.4 fold higher than in normal B cells (1 ± 0.1). These findings support recently published data showing that *FOXM1* message is elevated in DLBCL [38,39], and extend them to BL cell lines, suggesting that *FOXM1* deserves further consideration for the design and testing of new approaches to treat and prevent high-grade B lymphomas.

### Inhibitors that target FOXM1 reduce growth and survival of DLBCL and BL cells

**Figure 5:**
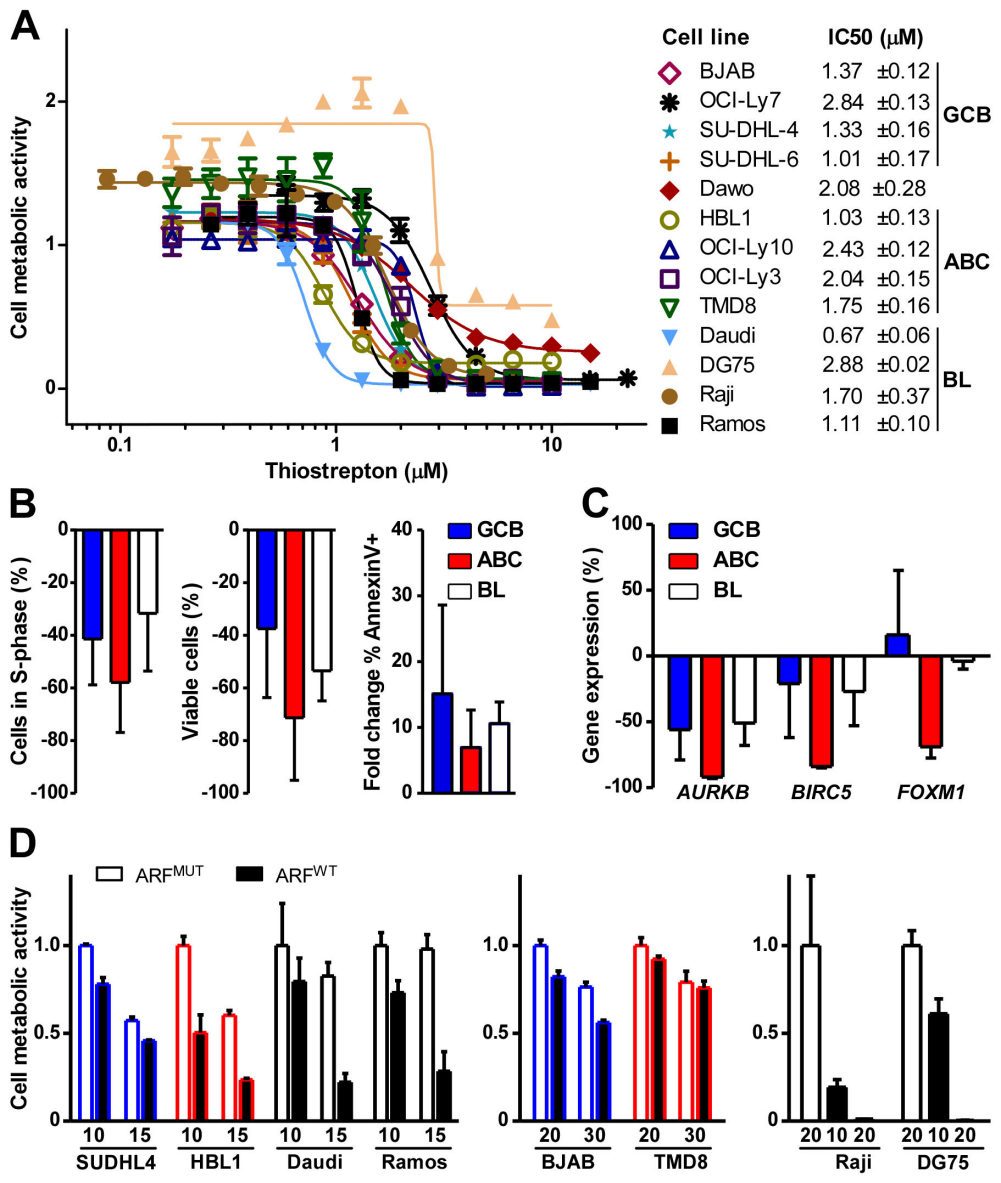
FOXM1 inhibitors impair growth and survival of DLBCL and BL cells *in vitro*. (A) Determination of the mean inhibitory concentration (IC_50_) of thiostrepton in 13 cell lines. Nine DLBCL and 4 BL cell lines were treated for 24 hrs with increasing concentrations of drug. Dose response curves are representative of at least three independent experiments. Cell metabolic activity was measured using the MTS assay. IC_50_ values and standard deviations are shown to the right. (B) Thiostrepton-dependent inhibition of cell proliferation and increased cell death. Tumor cell lines were exposed for 24 hrs to DMSO (vehicle control) or thiostrepton at IC_50_ levels shown in panel A, followed by flow cytometric determination of cells in S phase (DNA content analysis, left panel), number of viable cells (Guava ViaCount® analysis, middle panel), and apoptotic cells (annexin V immunoreactivity, right panel). Results are grouped by molecular subtype and shown as the average percent difference of the mean ± SD of thiostrepton- versus DMSO-treated cells. (C) Thiostrepton-dependent loss of gene expression. Cells were treated as described in panel B. RNA was isolated and gene transcript levels were measured using qPCR. Data are presented as described in panel B. (D) ARF peptide-dependent loss of cell metabolic activity. Four DLBCL and 2 BL cell lines were treated for 24 hrs with the indicated concentration (µM) of wild-type (WT) ARF peptide (black bars) or mutant (MUT) peptide (white bars) used as control. Cell metabolic activity was measured using the MTS (SUDHL4, BJAB, HBL1, TMD8) or CellTiter-Glo® (Daudi, Ramos, Raji, DG75) assays and is normalized to the mean of the lowest concentration of MUT peptide treatment per cell type. Blue, red and black outline colors indicate GCB, ABC, and BL cell lines, respectively. Data are representative of at least two independent experiments.

To evaluate the role of FOXM1 in growth and survival of neoplastic B-lymphocytes, DLBCL and BL cells were treated with thiostrepton, a thiazole antibiotic that has been shown to bind directly to FOXM1 and inhibit its activity [40]. Figure 5A shows that, in line with previous studies [39] thiostrepton was effective at low micromolar amounts in all 9 DLBCL lines we tested. Additionally thiostrepton was highly effective in 4 of 4 BL lines (Figure **5A**), with IC_50_ values ranging from 0.67 µM (Daudi) to 2.88 µM (DG75). Flow cytometry showed that treatment with thiostrepton resulted in cell cycle inhibition (Figure **5B** **left and** Figure **S2**), drop of viable cell numbers (Figure **5B** **center**), and increased apoptosis (Figure **5B** **right and** Figure **S3**). Expression of two of the 60 DMB genes, *AURKB* and *BIRC5*, has been reported to be positively regulated by FOXM1 [41], and we measured the mRNA level of these genes in cells treated with thiostrepton or vehicle control. Thiostrepton caused down regulation of *AURKB* and *BIRC5* in all cases (Figure **5C** **left and center**), with cells of the ABC subtype of DLBCL exhibiting the greatest reduction. ABC-DLBCL cells also demonstrated a dramatic reduction of *FOXM1* expression, which did not occur in GCB DLBCL and BL cells (Figure **5C** **right**). These results supported and extended recent findings on the inhibitory activity of thiostrepton in DLBCL cells [39] and revealed susceptibility of BL cells.

Because thiostrepton exhibits off-target effects (e.g., inhibition of proteasome activity [42]), we decided to confirm the results presented above using a different targeted agent. Working from knowledge that the tumor suppressor p14/p19^ARF^ binds and inhibits FOXM1 protein, the late Costa and his colleagues developed a cell penetrating peptide, ARF^WT^, that mimics ARF-dependent inactivation of FOXM1 [43]. A mutated form of the peptide that does not bind FOXM1, ARF^MUT^ is available as control. These peptides have been validated in multiple cell types [44]. Figure **5D** shows micromolar amounts of ARF^WT^ peptide inhibit growth and survival of DLBCL and BL cells. With the exception of TMD8, which was refractory for reasons that were not investigated (Figure **5D** **center**), to varying degrees all cell lines were sensitive to ARF^WT^ peptide at different doses. At higher concentrations, DLBCL cells exhibited non-specific toxicity to ARF^MUT^, but BL cells clearly demonstrated that ARF^WT^ was more effective than ARF^MUT^. The most dramatic response was observed in Raji and DG75 cells, in which treatment with equimolar amounts (20 µM) of ARF^MUT^ or ARF^WT^ left essentially all cells alive or metabolically dead (Figure **5D** **left**). The findings with thiostrepton and ARF^WT^ suggested that targeting FOXM1 may provide a therapeutic approach for patients with BL and DLBCL.

## Discussion

Key to our study was the use of Myc-dependent mouse B-cell lymphoma as a phylogenetically conserved filter for the analysis of the human DLBCL transcriptome. The gene expression changes in the mouse tumors permitted us to dramatically reduce the number of DLBCL candidates to the “DMB 60-gene” set. Genetic network analysis of this set resulted in the independent discovery of *FOXM1* as a putative lymphoma gene, providing evidence that our approach uncovered at least one known, biologically meaningful gene. Cox hazard regression analysis of the gene set linked the expression of 8 genes to survival of patients with DLBCL. Four of these genes were detected only because the mouse lymphoma transcriptome was available as a “biological filter” for gene expression analysis of human DLBCL. These results support the use of comparative genome-wide expression profiling of human-mouse lymphoma counterparts, complemented by functional genomics and clinical outcome studies, as a gene discovery tool that should also be considered for other types of B cell and plasma cell neoplasms.

Our finding that growth and survival of DLBCL and BL *in vitro* is reduced using agents known to target FOXM1 extends a growing body of evidence for a role of this forkhead protein as a promising target of cancer therapy [45]. Deregulated expression of FOXM1 results in centrosome amplification, mitotic catastrophe and other cytogenetic aberrations typically seen in cancer cells [46]. In normal cells, the level of FOXM1 is tightly regulated to ensure mitotic fidelity through the cell cycle [47]. In DLBCL, it was reported that *FOXM1* mRNA [30,31,33] and FOXM1 protein [39] are elevated [48], in part, because of genomic amplification that has been observed in about 50% (9 of 18) of DLBCL patients [38]. A level of FOXM1 above normal may provide a therapeutic threshold for targeted treatment. Further, targeted inhibition of FOXM1 either by siRNA or thiostrepton was recently reported to sensitize DLBCL cells to killing when combined with normally sub-toxic doses of the proteasome inhibitor bortezomib [39]. Thiostrepton did so despite the fact that like bortezomib it also functions as a proteasome inhibitor [42]. Using other drug combinations that include inhibition of FOXM1 may also be effective and testing is warranted in both DLBCL and BL.

The studies of Green et al. exposed an association between a *MYC* gene signature and MYC protein level with amplification of *FOXM1* in DLBCL [38]. It is well established that deregulated MYC is a prominent factor in DLBCL [49], and others have shown that FOXM1 is a target gene of MYC [50,51] and that MYC is a target gene of FOXM1 [52]. Using an unbiased, independent method, our cross-species analysis (notably with Myc as the initiating factor in mice) identified genetic networks that implicated both FOXM1 and MYC pathways. This supports the work of Green et al. and underscores the likelihood that MYC and FOXM1 support one another to drive or maintain aggressive B-lymphomas.

Several lines of evidence indicate that the DMB 60-gene list is of biological and clinical relevance. Twelve of the 60 DMB genes (20%) were confirmed in three independent GEP studies on aggressive B lymphoma. Six of these 12 genes (*CENPA, CKS1B, CKS2, LGALS3, NEK2, TOP2A*) may be of special interest because they have been the most frequently identified. *CENPA*, a regulator of kinetochore function, is upregulated upon transformation of B-lymphocytes with EBV [53]. *CKS1B* is involved in Myc-induced lymphoma in mouse and aggressive mantle cell lymphoma in humans [54]. *CKS2* governs replicative fidelity and cell cycle progression [55], and along with *CKS1B* and *NEK2*, may be a downstream target of FOXM1 [41]. *LGALS3*, the only anti-apoptotic member of the large galectin family of genes, regulates death in DLBCL cells [56]. *NEK2*, a regulator of mitosis [38] is a putative therapeutic target in patients with DLBCL [36]. Lastly, *TOP2A* encodes a direct target of doxorubicin, a drug in the standard CHOP regimen of lymphoma therapy. Improved understanding of the 6 genes described above, either individually or in concert, may lead to new approaches in treatment of high-grade B lymphoma.

Even as a potential target, FOXM1 expression did not predict overall survival in the Lenz et al. [18] study (CHOP: 1.024 HR, 0.792-1.325 95% CI; R-CHOP: 1.02 HR, 0.789-1.317 CI). However, eight of 60 (13%) DMB genes (*CCT3, CLCF1, COBLL1, CTPS, FABP5, HSPD1, MRPS17, NDC80*) did correlate with overall survival of DLBCL patients in that dataset. *HSPD1* encodes a heat-shock family protein chaperone that not only exhibits elevated expression in Hodgkin’s and large cell lymphoma [57] but also promotes the stability of the pro-survival factor BIRC5 (survivin; also a DMB 60-gene) [58]. *FABP5* encodes a fatty-acid binding protein and a target of MYC [59] that has been implicated in resistance of B-lymphoma cells to radiation [60]. None of the other genes have been implicated thus far in DLBCL or any B-lineage cancers. Despite one-third of the DMB 60-genes being associated with the cell cycle, only one of these eight genes, NDC80, has a known role in cell cycle activity during mitosis; it is implicated in cancer cell survival and kinetochore function [61]. *NDC80* was also identified as part of the expanded 6 gene network as shown in Figure 4C. CCT3, a subunit of the chaperonin complex that mediates appropriate folding of cytoskeletal proteins actin and tubulin, has also been implicated in cell division [62]. Perhaps a more provocative function of CCT3 is modulation of mRNA decay [63], changes in which could have broad implications on cell activity. The functions of the gene products of *COBLL1* or *MRPS17* are poorly characterized, making it difficult to speculate about their biological role if any in DLBCL. However, along with CTPS and FABP5, both have been associated with different aspects of cellular metabolism. COBLL1 has been associated with insulin sensitivity [64], MRPS17 is a mitochondrial ribosomal protein and might therefore regulate mitochondrial metabolic activity, CTPS is required for *de novo* cytosine biosynthesis, and FABP5 regulates intracellular fatty acid functions. Which of these myriad functions, if any, predominate to maintain or promote B-lymphomas remains to be determined. The only other gene of the eight, *CLCF1*, exhibited reduced expression in aggressive B-lymphomas. This is initially a bit counterintuitive, because CLCF1 is a cytokine that belongs to the IL-6 family and stimulates STAT3 activity [65], which is constitutively active in some DLBCLs [66]. Downregulation of CLCF1 transcription, however, could be the result of negative feedback inhibition involving STAT3 activation through alternative means. Importantly, four of the eight genes (*CLCF1, COBLL1, MRPS17, NDC80*) were not reproducibly deregulated in published GEP datasets on aggressive B lymphoma, and would have remained unrecognized without the benefit of the “mouse filter”.

In conclusion, our findings support the use of comparative gene expression profiling across species to identify candidate therapeutic targets in DLBCL and BL, and we unveiled several new potential biomarkers of DLBCL. Biological and clinical validation studies are warranted to evaluate these genes in greater depth, both as drivers of lymphoma development and therapeutic targets. 

## Supporting Information

Figure S1 **Graph showing gene expression profiling data variability using three dimensional principal component analysis (PCA) for both human (top) and mouse (bottom) samples.** Human control samples are blue and DLBCL tumor samples are red, whereas mouse control samples are red and tumor samples are blue.
(TIF)

Figure S2 **Representative DNA content histograms showing cell cycle distribution for DLBCL and BL cell lines treated with (+) and without (-** ) thiostrepton for 24h at the IC50 given in the text.
(TIF)

Figure S3 **Representative Annexin V staining dot plots and histograms for DLBCL and BL cell lines treated with (+) and without (-) thiostrepton for 24h at the IC50 given in the text.** Dot plots show restrictive gating for side and forward scatter profiles.
(TIF)

Table S1 **Sequences used for quantitative RT-PCR.**
(XLSX)

Table S2 **Concordant, differential probesets (n=183) and genes (n=130) in human DLBCL and mouse iMycBCL.**
(XLS)

Table S3 **Publicly available datasets used for proliferation comparison.**
(XLS)

Table S4 **DLBCL/iMyc genes also significantly differentially expressed (p≤0.01, 2-fold, Oncomine) in at least two other independent GEP studies.**
(XLS)

Table S5 **Cox regression analysis for association of DMB expression level with survival of DLBCL patients.**
(XLS)

Table S6 **STRING network analysis resulting from select DMB genes.**
(XLS)
