# Supplementary figures and images for: Identification of Candidate B-Lymphoma Genes by Cross-Species Gene Expression Profiling
Source: PLoS One. 2013 Oct 9;8(10):e76889. doi: 10.1371/journal.pone.0076889 (PMC3793908; doi:10.1371/journal.pone.0076889)

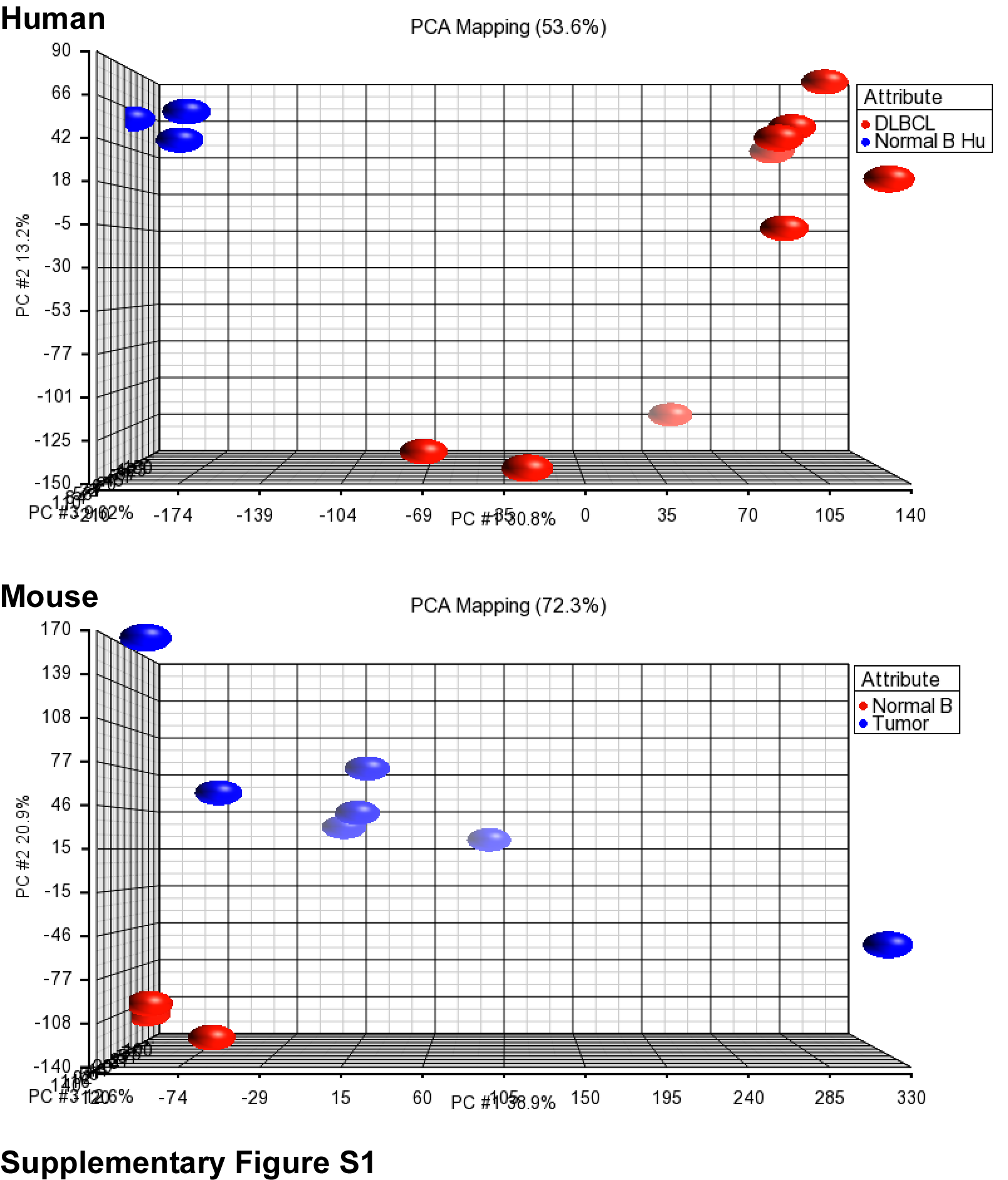

Supplement: Figure S1 — Graph showing gene expression profiling data variability using three dimensional principal component analysis (PCA) for both human (top) and mouse (bottom) samples. Human control samples are blue and DLBCL tumor samples are red, whereas mouse control samples are red and tumor samples are blue. (TIF) [file pone.0076889.s001.tif]

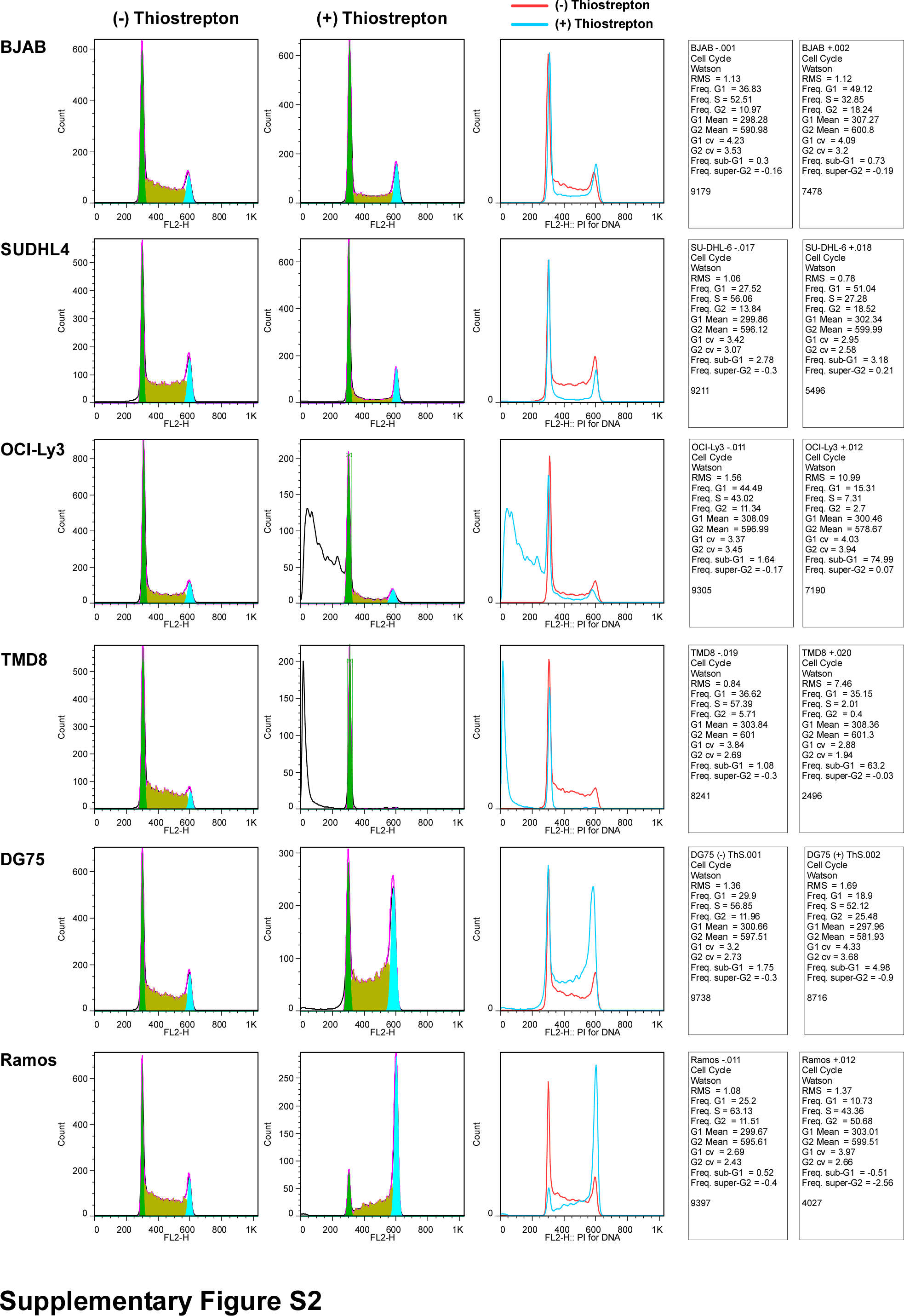

Supplement: Figure S2 — Representative DNA content histograms showing cell cycle distribution for DLBCL and BL cell lines treated with (+) and without (- ) thiostrepton for 24h at the IC50 given in the text. (TIF) [file pone.0076889.s002.tif]

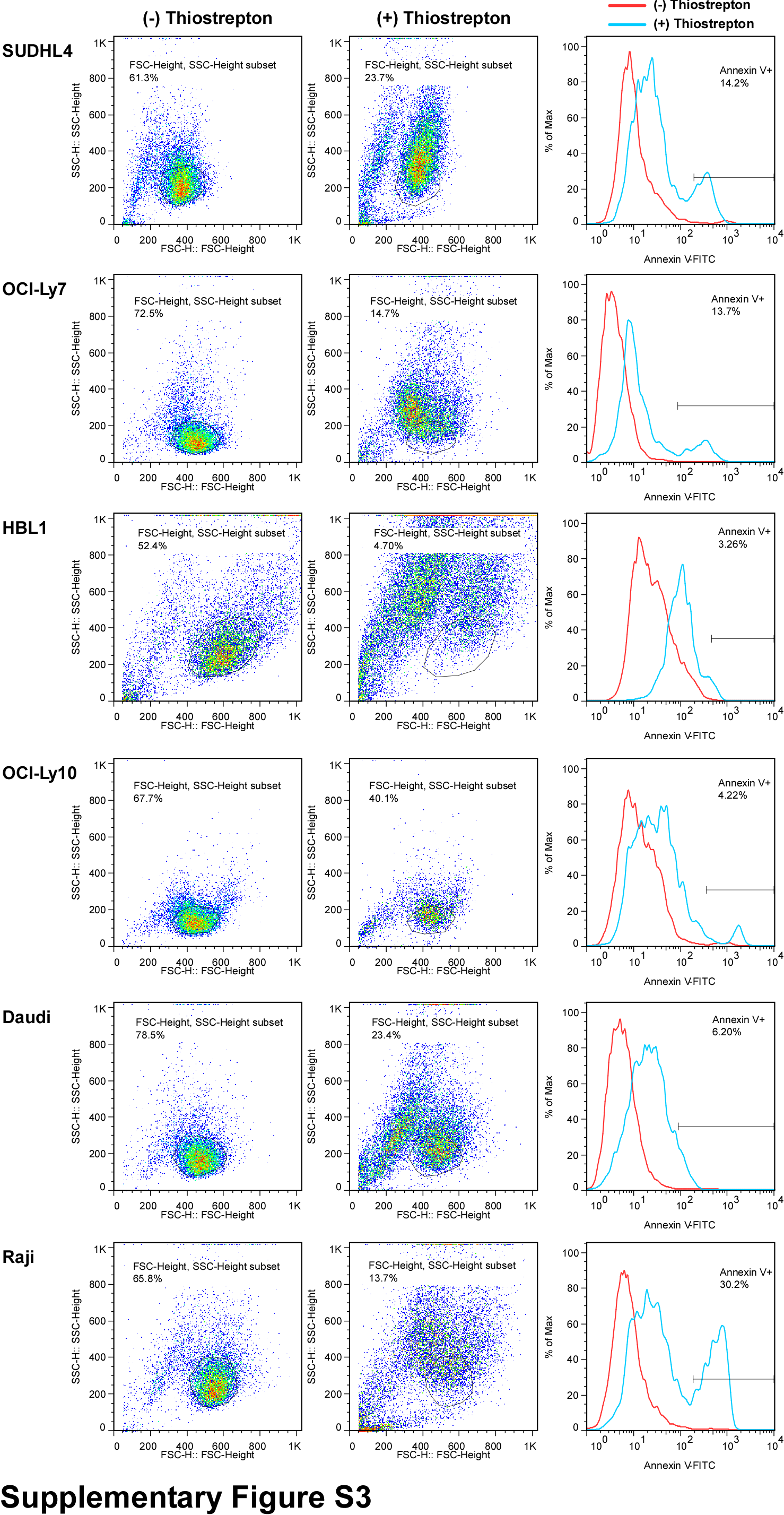

Supplement: Figure S3 — Representative Annexin V staining dot plots and histograms for DLBCL and BL cell lines treated with (+) and without (-) thiostrepton for 24h at the IC50 given in the text. Dot plots show restrictive gating for side and forward scatter profiles. (TIF) [file pone.0076889.s003.tif]
